# The STIIM UP Protocol: Exploratory Evaluation of the Potential of Combined Calcium Hydroxyapatite, Hyaluronic Acid, and Botulinum Toxin A in Neck Rejuvenation

**DOI:** 10.3390/jcm15156052

**Published:** 2026-08-04

**Authors:** Valéria Dal Col, Cassiano Santos Marchi, Maria Auxiliadora Dinalli Marchi, Barbara Barquette Silva da Rosa, Beatriz Domenici de Oliveira, Natália de Magalhães Ferreira, Fábio Fernandes Ribas, Renata Viana

**Affiliations:** 1 Protocoll Clinique, Vitória 29055-145, ES, Brazil; fabioribas@gmail.com; 2 Private Practice, Santo André 09080-511, SP, Brazil; casmar@bol.com.br (C.S.M.); dramariadinalli@gmail.com (M.A.D.M.); 3 Private Practice, São Paulo 04101-000, SP, Brazil; barbara_barquette@hotmail.com; 4 Ilikia, São Paulo 01427-000, SP, Brazil; beatriz.domenici.oliveira@gmail.com (B.D.d.O.); renata27@gmail.com (R.V.); 5 Private Practice, Belo Horizonte 30380-403, MG, Brazil; nataliamagalhaesferreira@gmail.com

**Keywords:** calcium hydroxyapatite, hyaluronic acid, botulinum toxin type A, skin biostimulation, neck rejuvenation

## Abstract

**Background/Objectives:** Cervical integument aging is a complex, multifactorial process characterized by thinning dermis, loss of elasticity, and platysmal band prominence. Multimodal treatments targeting these distinct anatomical layers simultaneously remain limited. This study aimed to evaluate the clinical efficacy, safety, and rejuvenation potential of the STIIM UP Protocol, a single-session treatment combining calcium hydroxyapatite (CaHA), hyaluronic acid (HA), and botulinum toxin type A (BoNT-A) for neck rejuvenation. **Methods:** This prospective, open-label, exploratory clinical study enrolled 13 healthy women (n = 13) presenting with cervical laxity. The STIIM UP suspension was delivered bilaterally into the subdermal plane of the anterior neck region using a retro-injection fanning technique with a 22G cannula at Day 0 (D0). Evaluated outcomes included the IBSA Neck Grading Scale, Global Aesthetic Improvement Scale (GAIS), FACE-Q module scores, and high-frequency ultrasound (USG) tissue thickness measurements at baseline and Day 120 (D120). Safety was monitored throughout. **Results:** The median consensus IBSA scores decreased significantly from 4.0 at baseline to 2.0 at both D30 and D120 (*p*-value < 0.01), indicating a rapid and sustained reduction in laxity. At D120, the GAIS responder rate reached 100% according to independent evaluators and 90.9% based on patient self-assessments. Quantitative USG confirmed statistically significant bilateral tissue thickening across the epidermis, dermis, and subcutaneous strata (*p*-value < 0.05). Progressive, clinically meaningful improvements were captured via the FACE-Q scales. No local or systemic adverse events were observed. **Conclusions:** The STIIM UP Protocol is a safe and effective multilayer intervention that successfully achieves comprehensive neck rejuvenation, bridging immediate clinical enhancement with sustained, tissue-proven structural remodeling.

## 1. Introduction

The aging of cervical integument (neck skin) is a progressive and multifactorial process marked by loss of elasticity, thinning dermis, and muscle relaxation [1,2,3]. It results from intrinsic (genetic) and extrinsic (UV/sun) factors, manifesting as sagging, wrinkles (rhytides), and prominent platysmal bands, often appearing faster than facial aging due to thinner skin [1,2,3]. Anatomically, this vulnerability is driven by a unique architecture wherein the cervical dermis is approximately 40% thinner than that of the face [1,2,3,4]. Furthermore, a significantly lower density of sebaceous glands results in a compromised hydrolipidic film, thereby reducing innate barrier function and increasing susceptibility to environmental oxidative stress [1,2,3,4]. Consequently, significant clinical manifestations of aging are frequently observed as early as the third decade of life [1,2,3,4].

Historically, a diverse array of therapeutic modalities has been employed to address cervical aging, spanning non-invasive, minimally invasive, and surgical interventions. Non-invasive approaches frequently rely on energy-based devices (EBDs)—such as fractionated or bipolar radiofrequency (RF) and high-intensity focused ultrasound (HIFU)—which deliver controlled thermal energy to the deep dermis and fibromuscular layers to induce tissue contraction and subsequent neocollagenesis [5,6,7]. Minimally invasive mechanical modalities, including microneedling and structural suspension threads, are also utilized to physically stimulate skin repair mechanisms or mechanically reposition lax tissues [8,9]. Within the domain of injectables, conventional monotherapies have historically focused on compartmentalized issues; for instance, the administration of uncrosslinked or low-crosslinked hyaluronic acid (HA) “skin boosters” primarily addresses superficial dermal hydration and the filling of static horizontal rhytides, whereas standalone botulinum toxin type A (BoNT-A) injections specifically target muscular hyperfunctional states, such as prominent platysmal bands [10,11]. Despite their utility, these single-modality protocols often present limitations, frequently requiring multiple treatment sessions, demonstrating variable longevity, or failing to comprehensively address the simultaneous structural degradation of the epidermal, dermal, and muscular layers of the aging neck.

Contemporary therapeutic strategies have shifted from monotherapies toward multimodal approaches, frequently combining the biostimulation of calcium hydroxyapatite (CaHA) with other modalities, such as neurotoxins and hyaluronic acid (HA) fillers, in a single session to achieve comprehensive skin tightening and remodeling [4,12,13,14,15,16].

CaHA is a well-established, biocompatible, and biodegradable biostimulator that promotes neocollagenesis and improves dermal structural integrity [16,17,18,19,20,21]. Complementarily, HA provides immediate structural support and hydration through its high hygroscopic capacity [22,23,24,25]. Furthermore, the integration of botulinum toxin type A (BoNT-A) addresses the muscular component of aging by relaxing the platysma muscle, thereby smoothing dynamic bands and refining the cervical contour [26,27,28,29].

While the individual benefits of these agents are documented [16,22,23,30], clinical data regarding their combined application for neck rejuvenation remains limited to scale-based observational outcomes [30]. Thus, this study aimed to evaluate the clinical efficacy, patient satisfaction, and regenerative potential of a combined protocol (STIIM UP) involving CaHA (STIIM^®^) and HA (UP Fine^®^), both manufactured by CG Bio Co., Ltd. (Seoul, Republic of Korea), and BoNT-A.

## 2. Materials and Methods

### 2.1. Study Design and Ethics

This was a prospective, open-label, single-arm clinical study conducted at a single research center in Brazil over a period of 120 days. The study was performed in accordance with the Declaration of Helsinki and Good Clinical Practices (GCP), with prior approval from the Institutional Research Ethics Committee of the Escola Superior de Ciências da Santa Casa de Misericórdia de Vitória (EMESCAN, CAAE number 88557325.7.0000.5065). All participants provided written informed consent before undergoing any study-related procedures.

### 2.2. Study Population

A total of 13 healthy women (*N* = 13), all older than 35 years, with clinically evident neck laxity confirmed by the 5-point IBSA Neck Grading Scale [31], and a stable Body Mass Index (BMI) below 25 kg/m^2^ were included in the study. Participants were eligible for inclusion only if they demonstrated a clear understanding of the study procedures and agreed to refrain from undergoing any additional esthetic treatments in the neck throughout the study period. They were also required to commit to attending all scheduled follow-up visits and to adhere to all study-related instructions and responsibilities. Subjects were excluded from participation if they had hypersensitivity to the study products, received previous neck treatments within the last 24 months, were pregnant or lactating, or had active infections or severe scarring in the treatment area. Recruitment occurred during routine clinical practice by the research team. Individuals who met the eligibility criteria and expressed interest were invited to participate.

The decision to conduct this exploratory clinical trial with a sample of 13 participants was scientifically and statistically justified given the study’s primary objective: to generate preliminary safety and efficacy signals for the combined use of CaHA, HA, and BoNT-A. Early-phase esthetic and dermatologic intervention studies commonly employ small cohorts because they aim to characterize treatment feasibility, optimize protocols, and estimate effect sizes rather than test definitive hypotheses. A sample of 10–20 participants is widely accepted in pilot research as sufficient to detect consistent trends, identify adverse events, and calculate variance estimates needed for powering subsequent randomized trials [32,33]. In this context, a cohort of 13 participants provides adequate precision for within-subject comparisons, supports the detection of clinically meaningful changes across standardized outcome measures, and minimizes unnecessary exposure to novel combination therapies before robust safety data are established. This sample size therefore aligns with methodological standards for exploratory investigations while ensuring ethical stewardship of participant risk.

### 2.3. Investigational Products and Preparation

The protocol utilized a combination of three primary agents in a single syringe to achieve a combined suspension:

- CaHA (STIIM^®^; 1.5 mL): A commercial dermal implant, approved by Brazilian Health Regulatory Agency (Anvisa, registry number 10378640018), composed of 30% synthetic calcium hydroxyapatite microspheres suspended in a 70% carboxymethylcellulose gel carrier. STIIM^®^ is produced by CG Bio Co., Ltd. (Republic of Korea).
- HA (UP Fine^®^; 2 mL): A commercial filler composed of hyaluronic acid gel at a concentration of 20 mg/mL, integrated with 0.3% lidocaine hydrochloride to mitigate procedural discomfort; it was approved by Anvisa (registry number 10378640017). UP Fine^®^ is produced by CG Bio Co., Ltd. (Republic of Korea).
- BoNT-A (Botulift^®^): Commercial neuromuscular modulators produced by Medytox, Inc. (Cheongju-si, Republic of Korea).
- Diluent: An additional amount of 2% lidocaine to reach a total volume of 6 mL. Lidocaine is included in the injectable mixture to ensure that the patient does not experience discomfort during the procedure, providing an additional layer of analgesia even when local anesthesia has already been administered.

The final suspension (1.5 mL STIIM^®^, 2 mL UP Fine^®^, 20 U BoNT-A and diluent) in a total volume of 6 mL used in this study was designated as the STIIM UP Protocol.

### 2.4. Clinical Protocol (Treatment)

**Figure 1:**
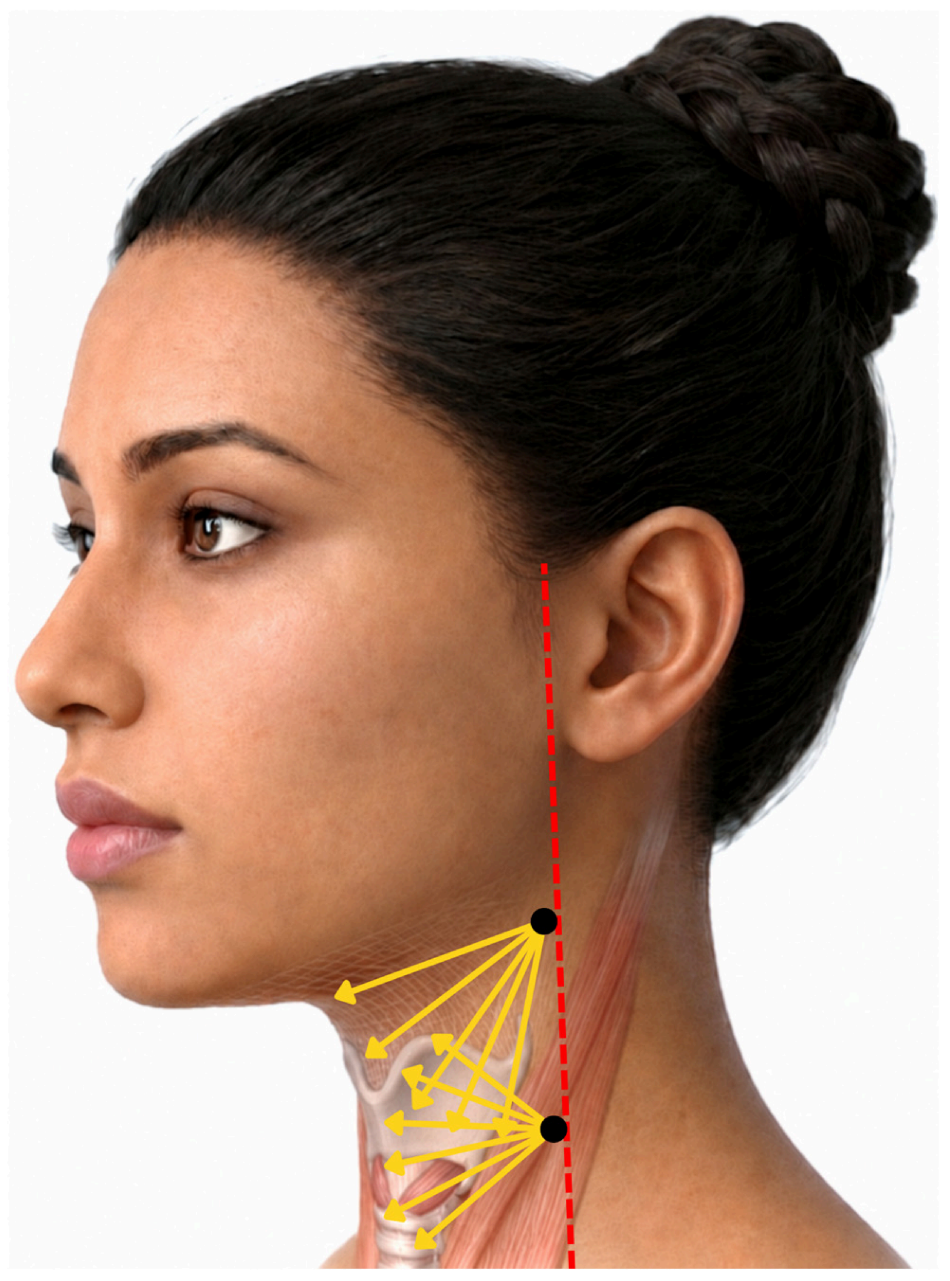
Retro-injection fanning technique for submental and cervical rejuvenation. This figure illustrates the retro-injection fanning technique used for the delivery of dermal fillers or biostimulators in the submental and upper cervical regions. Two primary injection ports (indicated by black dots) are positioned along the posterior margin of the treatment area, typically aligned with the anterior border of the sternocleidomastoid muscle (indicated by the red dashed line). The yellow arrows represent the fanning pattern of the cannula. From a single-entry point, the cannula is advanced to its distal extent, and the product is deposited in a linear thread as the cannula is withdrawn (retrograde injection). Image generated using Gemini AI.

Following aseptic preparation and local anesthesia, the STIIM UP Protocol was delivered to the latero-anterior cervical region using a 22G × 70 mm cannula. The product was administered bilaterally, with 3 mL per entry point, into the subdermal plane using a retro-injection fanning technique to ensure homogeneous distribution at Day 0 (D0), as illustrated in Figure 1. Post-application massage was performed to optimize uniform product placement.

All participants were explicitly instructed not to undergo any energy-based cosmetic procedures (including radiofrequency, ultrasound, and laser treatments) or any additional esthetic interventions, such as facial or neck massage, during the entire study period.

### 2.5. Evaluation Methods

#### 2.5.1. Scale-Based Efficacy Outcomes

Efficacy outcomes were assessed using a combination of validated esthetic evaluation tools to capture both clinician-reported and patient-reported improvements. Objective clinical changes in cervical contour were quantified through the IBSA Neck Grading Scale [31], which provided a standardized assessment of neck laxity and definition. Neck esthetic improvement was further evaluated by investigators and participants using the Global Aesthetic Improvement Scale (GAIS) [34]. Patient-reported satisfaction and perceived benefit were measured through the FACE-Q modules *Satisfaction with Outcome* and *Neck*, allowing for a comprehensive evaluation of treatment impact on appearance-related quality of life [35].

Standardized photographic documentation was obtained immediately before treatment with STIIM UP Protocol on Day 0 (D0) to enable evaluation using the IBSA Neck Grading Scale. This imaging protocol, along with all scale-based assessments, was repeated after 30-day and 4-month intervals throughout the follow-up period to monitor progressive changes over time. Assessments were therefore conducted on Day 30 (D30) and Day 120 (D120), which corresponds to the study endpoint. This schedule allowed for consistent longitudinal comparison of neck contour improvements and treatment durability.

#### 2.5.2. Ultrasound Analysis

Skin evaluation was performed using high-frequency ultrasound (US) with the Evus 5 device (Saevo, Ribeirão Preto, Brazil), following the methodology previously described by our group [17]. US assessments were conducted immediately before treatment initiation and at the study endpoint (D120). A 16 MHz linear transducer was positioned directly over the region of interest using acoustic coupling gel to ensure optimal contact and minimize air interference. This high-frequency probe provides enhanced axial and lateral resolution, enabling detailed, non-invasive visualization of the epidermis, dermis, and subcutaneous layers. Bilateral neck imaging (right and left sides) was performed for all participants. The thickness (mm) of the epidermis, dermis, and subcutaneous tissues was measured and analyzed.

**Figure 2:**
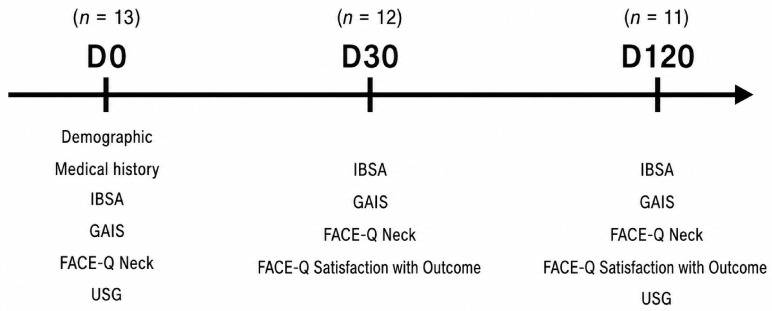
Schematic representation of the clinical trial across the 120-day follow-up period. The trial assessed a cohort starting at Day 0 (*n* = 13), with subsequent follow-up evaluations at Day 30 (*n* = 12) and Day 120 (*n* = 11). Baseline assessments at Day 0 (D0) included patient demographics and medical history. Clinical, patient-reported, and diagnostic outcomes were tracked across specific time points using the IBSA scale, Global Aesthetic Improvement Scale (GAIS), FACE-Q Neck Scale, FACE-Q Satisfaction with Outcome Scale, and ultrasound (USG).

The activities conducted throughout the study were systematically organized and are presented in Figure 2, which shows the complete study protocol. This figure details the sequence, duration, and timing of each methodological step, ensuring transparency and allowing for clear visualization of the workflow adopted during the research process.

### 2.6. Safety Monitoring

The clinical application of the combined suspension involves safety considerations related to both the specific pharmacological properties of the constituents and the mechanics of the injection procedure itself. Potential adverse effects associated with the individual products include local hypersensitivity or allergic reactions, prolonged tenderness, and the formation of palpable nodules or granulomas as the carboxymethylcellulose carrier for the calcium hydroxyapatite (CaHA) degrades and tissue biostimulation occurs. Regarding the delivery method, the use of a 22G cannula for deep subdermal retrograde fanning injections carries inherent procedural risks, such as temporary pain at the injection site, localized erythema, transient edema, ecchymosis, exacerbated inflammatory reactions, and localized infection.

Despite these potential risks, no local or systemic adverse events were observed in any of the patients throughout the entire 120-day follow-up period. Expected early post-procedural sequelae, such as mild transient swelling or minor bruising, completely resolved well within the standard 15-day baseline window and did not meet the criteria for classification as adverse events. Expected local reactions, such as edema or ecchymosis, were only considered AEs if they persisted beyond 15 days.

### 2.7. Statistical Analysis

Descriptive statistical methods were used to characterize the study population at baseline. Continuous demographic and clinical variables, including age, weight, height, and BMI, were summarized using means and standard deviations, with corresponding 95% confidence intervals to quantify precision. Categorical variables, such as race, medical history, and prior esthetic procedures, were reported as frequencies and percentages. The results were summarized in a descriptive table.

Statistical analyses focused on evaluating inter-rater reliability and longitudinal changes in IBSA scores. Inter-rater agreement among the three evaluators was assessed independently at each study visit (D0, D30, and D120) using pairwise weighted Cohen’s kappa coefficients with squared weights to account for the ordinal structure of the 5-point scale. For efficacy analyses, evaluator ratings were aggregated by computing the median IBSA score per subject and visit, providing a consensus measure robust to individual rater variability. Descriptive statistics for IBSA outcomes were reported as median values with interquartile ranges and bootstrap-derived 95% confidence intervals (5000 resamples). Longitudinal changes were examined using paired Wilcoxon signed-rank tests for the D0–D30, D30–D120, and D0–D120 intervals, with Holm correction applied to control the family-wise error rate. Effect sizes were quantified using the rank-biserial correlation. To further characterize treatment response, individual score changes (ΔIBSA) were summarized descriptively, and the proportion of participants achieving at least a one-point improvement was estimated using exact binomial methods with corresponding 95% confidence intervals.

The GAIS was evaluated as an ordinal categorical endpoint at D30 and D120 using ratings from three independent evaluators and from patient self-assessment, preserving the ordered structure of the five GAIS categories (“Very Much Improved”, “Much Improved”, “Improved”, “No Change”, and “Worse”). Inter-rater agreement at each visit was quantified using weighted Cohen’s kappa coefficients with squared weights to account for the magnitude of disagreement across ordinal levels, with pairwise comparisons performed among all evaluator combinations and between each evaluator and the patient. The dataset was then reshaped into a long format to enable descriptive analyses of category-specific frequency distributions by visit and rater. Responder analyses were conducted by dichotomizing GAIS into responder (“Improved” or better) versus non-responder (“No Change”), and responder proportions with corresponding 95% confidence intervals were estimated for each evaluator and patient assessment at each visit using exact binomial tests.

FACE-Q Neck scores were evaluated longitudinally using within-subject change scores derived from paired assessments at baseline (D0), Day 30 (D30), and Day 120 (D120), whereas FACE-Q Satisfaction with Outcome scores were evaluated using paired post-treatment assessments at D30 and D120. For each participant, deltas were computed for all visit contrasts (D30–D0, D120–D30, and D120–D0), and the distributional properties of these change scores were examined using the Shapiro–Wilk test; no comparison demonstrated significant deviation from normality (all *p* > 0.05), supporting the use of parametric inference. Descriptive analyses included the mean, standard deviation, and 95% confidence intervals for both absolute FACE-Q Neck and Satisfaction with Outcome scores and their corresponding longitudinal changes. Inferential analyses were performed using paired Student’s *t*-test, with effect sizes quantified via paired Cohen’s *d*. To account for multiplicity across the three planned pairwise contrasts, *p*-values were adjusted using the Holm procedure.

Longitudinal changes in epidermal, dermal, and subcutaneous tissue thickness between baseline (D0) and Day 120 (D120) were evaluated utilizing a non-parametric framework, necessitated by the violation of normality assumptions across several delta distributions, as determined by the Shapiro–Wilk test. Descriptive statistics for continuous variables are expressed as medians, interquartile ranges (IQRs), quartiles, and bootstrap-derived 95% confidence intervals (CIs) for the medians. To maintain statistical integrity, analysis was restricted to complete paired cases, excluding participants presenting incomplete bilateral data for the specific side under evaluation. Intra-subject comparisons between time points (D0 versus D120) were performed using the paired Wilcoxon signed-rank test, and the magnitude of longitudinal shifts was quantified using Wilcoxon effect size statistics (r). All statistical tests were two-tailed, with the threshold for statistical significance set at 5%.

## 3. Results

### 3.1. Baseline Demographic and Clinical Profile

The study enrolled thirteen female participants with a mean age of 48.5 ± 7.5 years, mean weight of 56.6 ± 7.9 kg, mean height of 159.4 ± 6.6 cm, and mean BMI of 22.2 ± 2.0 kg/m^2^ (Table 1). Most subjects were white (84.6%), and the cohort was generally healthy, with nine participants (69.2%) reporting no relevant medical history and four (30.8%) reporting smoking and/or other medical conditions (Table 1). Prior exposure to esthetic procedures was limited: four subjects (30.8%) had previously undergone cosmetic treatments, including botulinum toxin injections (subjects 2, 4, and 13), hyaluronic acid fillers (subjects 2 and 13), microneedling (subject 10), and biostimulation therapy (subject 13). Overall, the sample represented a relatively healthy, middle-aged female population with minimal prior cosmetic interventions.

### 3.2. STIIM UP Protocol Promotes Sustained Reduction in Neck Laxity Across the 120-Day Follow-Up

The inter-rater reliability for the IBSA scale varied across evaluator pairs and study visits, ranging from slight-to-moderate to substantial agreement. At baseline (D0), the weighted Cohen’s kappa coefficients ranged from 0.295 to 0.952, reflecting fair concordance between Evaluators 1 and 2 (κ = 0.295), fair concordance between Evaluators 1 and 3 (κ = 0.360), and almost perfect agreement between Evaluators 2 and 3 (κ = 0.952, Table A1). Agreement was more heterogeneous at D30 (κ = 0.063–0.556), but improved by D120, with coefficients ranging from 0.522 to 0.790, indicating moderate-to-substantial concordance (Table A1).

**Figure 3:**
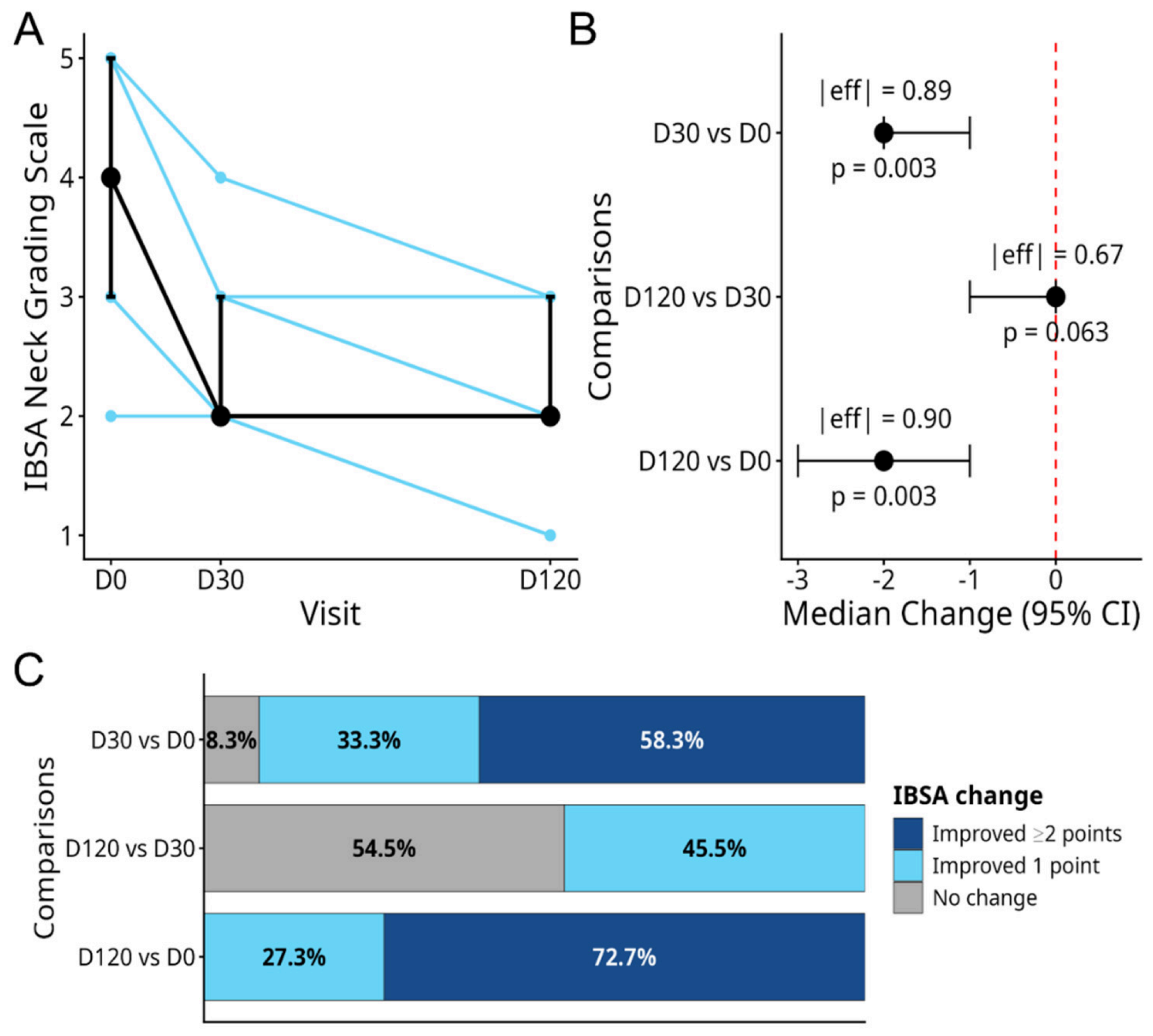
Longitudinal analysis of IBSA scores. (**A**) Individual IBSA score trajectories across visits (blue lines), overlaid with the median consensus score at each time point (black line) and corresponding 95% bootstrap confidence intervals (vertical black bars). The plot illustrates a rapid reduction in IBSA scores by Day 30, followed by stabilization through to Day 120. (**B**) The plot displays the median change in IBSA scores (black circle) and the 95% CI (horizontal black line). Collectively, the pattern demonstrates a clear, time-dependent reduction in IBSA scores, with the greatest improvement occurring by Day 30 and maintained through to Day 120. Eff represents the effect size, and the red line represents the absence of an effect. Negative results indicate IBSA scale improvement. (**C**) Proportion of subjects that showed IBSA improvement, confirming that the STIIM UP Protocol promoted a time-dependent improvement in IBSA scale score.

The median consensus IBSA scores demonstrated a marked reduction from 4.0 (IQR = 2.0; 95% CI, 3.0–5.0) at baseline to 2.0 at both D30 (IQR = 1.0; 95% CI, 2.0–3.0) and D120 (IQR = 0.5; 95% CI, 2.0–3.0, Table 2, Figure 3A).

Paired comparisons revealed significant improvements from D0 to D30 (Wilcoxon signed-rank *p*-value = 0.00098; Holm-adjusted *p*-value = 0.00293; effect size (r) = 0.896) and from D0 to D120 (*p*-value = 0.00098; Holm-adjusted *p*-value = 0.00293; effect size (r) = 0.897), both with large effect sizes (Table 3, Figure 3B). No significant change was observed between D30 and D120 (*p*-value = 0.0625), although the effect size remained large (effect size (r) = 0.674), indicating stabilization rather than further improvement (Table 3, Figure 3B).

Analysis of individual score trajectories supported these findings: 91.7% of subjects (11/12; 95% CI, 61.5–99.8%) improved by at least one point between D0 and D30, with a median ΔIBSA of −2 (IQR = 1; Table 4, Figure 3C). Between D30 and D120, 45.5% (5/11; 95% CI, 16.7–76.6%) exhibited additional improvement, whereas the median change was 0 (IQR = 1; Table 4, Figure 3C). From baseline to D120, all evaluable subjects (11/11; 95% CI, 71.5–100.0%) achieved at least a one-point reduction, with a median improvement of −2 points (IQR = 1; Table 4, Figure 3C). Collectively, these findings demonstrate a rapid and clinically meaningful reduction in neck laxity by Day 30 that was maintained through to Day 120 (Figure 3C).

**Figure 4:**
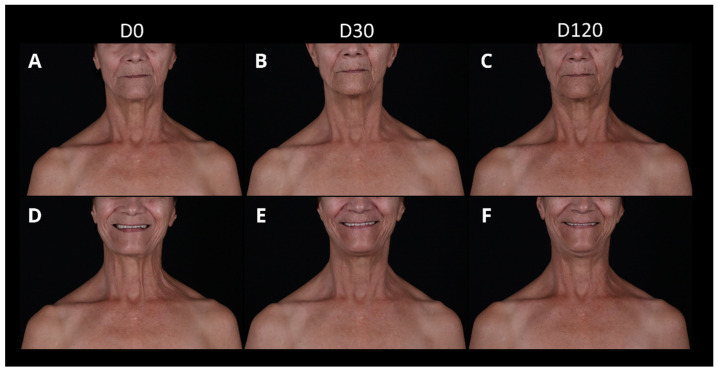
Clinical evolution of neck rejuvenation using the STIIM UP Protocol. Representative photographic records of a female patient at baseline (D0; (**A**,**D**)), 30 days post-treatment (D30; (**B**,**E**)), and 120 days post-treatment (D120; (**C**,**F**)) with neutral (top row) and dynamic/smiling (bottom row) expressions. At D30, a noticeable improvement in skin texture, horizontal lines, and platysmal bands is evident, reflecting a reduction in scores on the IBSA Neck Laxity Scale and a corresponding increase in patient-reported satisfaction on the FACE-Q Satisfaction with Outcome scale. These clinical improvements and structural gains were visibly maintained at the long-term follow-up at D120. The participant provided written informed consent for the publication and dissemination of these images.

As illustrated in Figure 4, the application of the STIIM UP Protocol resulted in a visible reduction in superficial wrinkling and an enhancement of skin firmness in the neck region at 30 days (D30), with sustained therapeutic efficacy and maintenance of the clinical improvements observed at 120 days (D120) post-intervention.

### 3.3. GAIS Outcomes Reveal Improvement

**Figure 5:**
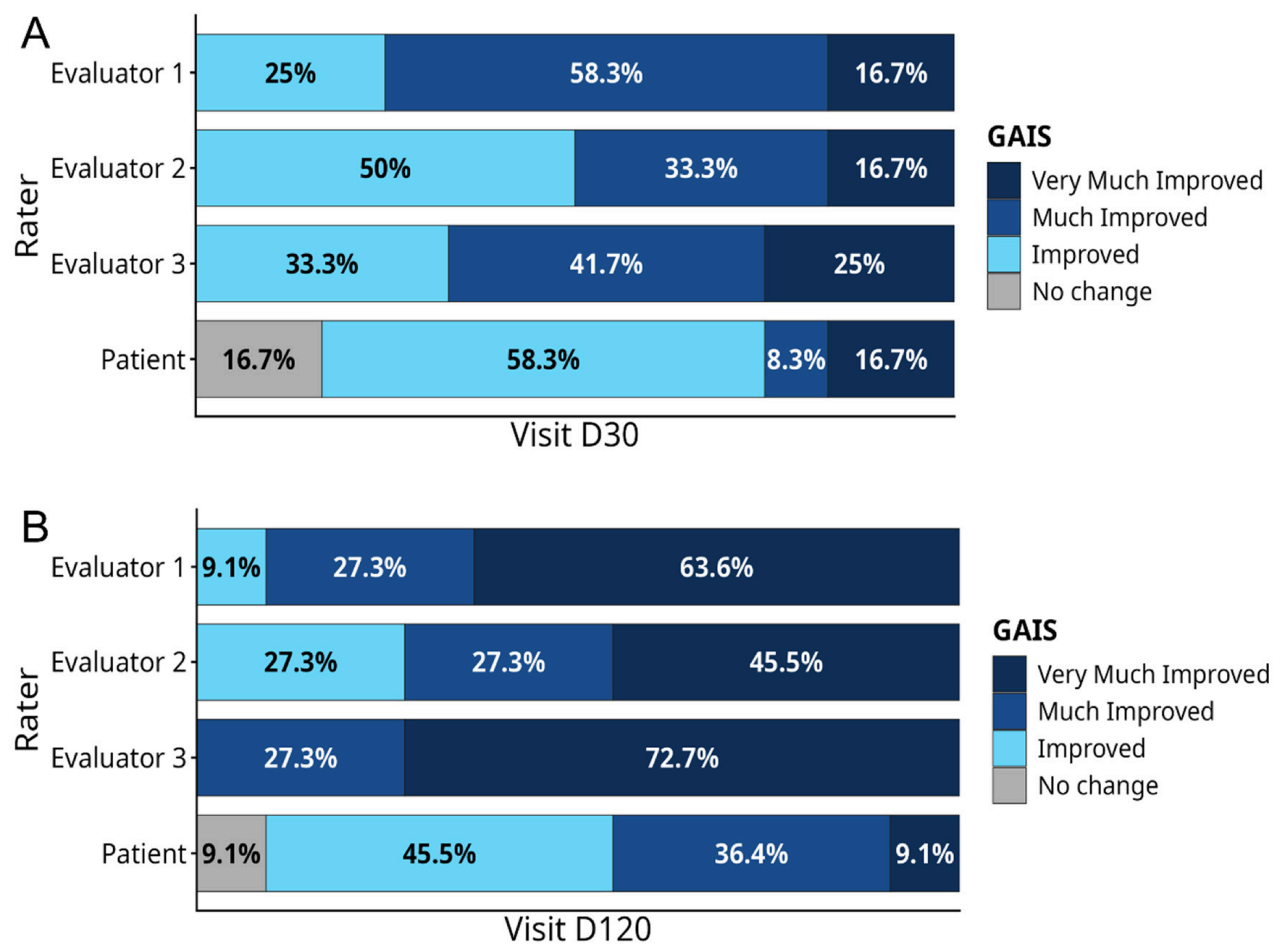
Evaluator and patient GAIS ratings at Day 30 (**A**) and Day 120 (**B**). (**A**) At Day 30 (D30), evaluator assessments were predominantly clustered within the “Improved” to “Much Improved” categories: Evaluator 1 classified 58.3% of subjects as “Much Improved”, Evaluator 2 classified 50.0% as “Improved”, and Evaluator 3 classified 41.7% as “Much Improved”. Patient self-assessments at D30 were more conservative but remained strongly positive, with 83.3% (10/12) reporting at least some improvement, including 16.7% rating themselves as “Very Much Improved”. (**B**) By Day 120 (D120), evaluator-perceived improvement intensified, with 63.6%, 45.5%, and 72.7% of subjects rated as “Very Much Improved” by Evaluators 1, 2, and 3, respectively. Patient ratings again trended more modestly, with most participants reporting either “Improved” (45.5%) or “Much Improved” (36.4%). The figure illustrates the divergence in absolute rating distributions between evaluators and patients, while highlighting the overall consistent perception of esthetic benefit over time.

The inter-rater agreement for GAIS was low at both D30 and D120, indicating variability among evaluators in the categorical grading of improvements (Figure 5A). Despite this variability, most assessments indicated esthetic improvement. At D30, 100% of subjects were classified as at least “Improved” by all three independent evaluators (12/12), while 83.3% of patients (10/12) rated themselves as at least “Improved” (Figure 5A). At D120, this proportion remained 100% (11/11) for all three evaluators, and among patient-reported responses, it increased to 90.9% (10/11, Figure 5B). These findings suggest a consistent perception of esthetic benefit, although the exact magnitude of improvement varied among raters. The inter-evaluator statistical analysis is shown in Table A2 and Table A3.

Responder analyses demonstrated uniformly high rates of improvement across evaluators. At D30, all evaluators classified 100% of subjects (12/12) as responders (“Improved” or better), corresponding to a response rate of 100% (95% CI: 73.5–100.0%, Table 5). The patient-reported response rate at D30 was 83.3% (10/12; 95% CI: 51.6–97.9%, Table 5). At D120, the evaluator-rated response rate remained 100% (11/11; 95% CI: 71.5–100.0%), while the patient-reported response rate increased to 90.9% (10/11; 95% CI: 58.7–99.8%, Table 5). Overall, although GAIS ratings exhibited substantial inter-rater variability, both evaluators and patients consistently perceived meaningful esthetic improvement, with evaluator assessments indicating a progressive enhancement in appearance over time.

### 3.4. STIIM UP Protocol Sustained Longitudinal Gains in Patient-Reported FACE-Q Neck Outcomes

**Figure 6:**
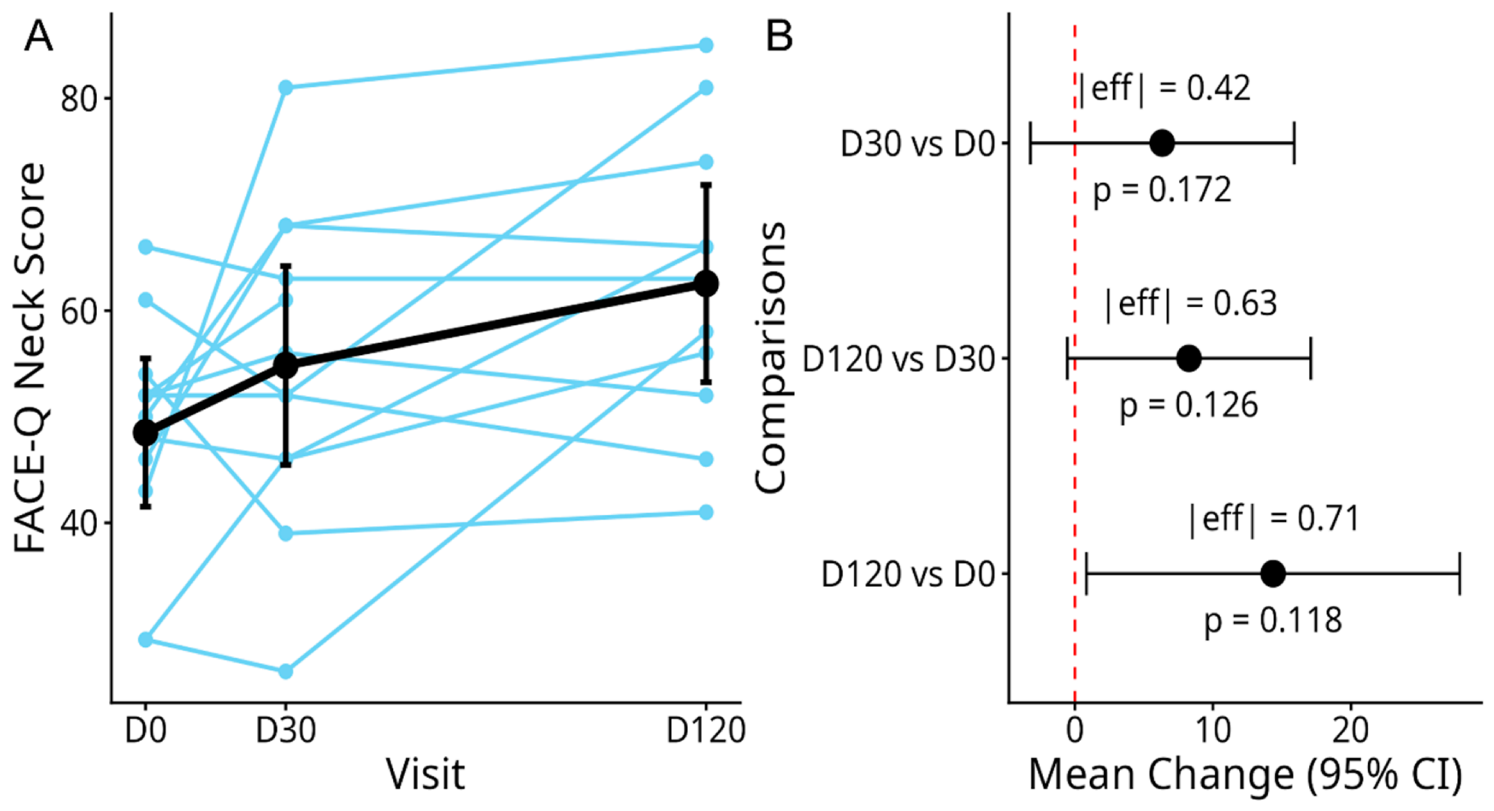
Evolution of FACE-Q Neck scores and pairwise comparisons across the study period. (**A**) FACE-Q Neck scores progressively increased over the study period, indicating sustained improvement in patient-reported neck appearance following treatment. Light blue lines represent individual patient trajectories across visits on Day 0 (D0), Day 30 (D30), and Day 120 (D120). The solid black line and error bars represent the mean score and its corresponding 95% confidence interval (95% CI). (**B**) Forest plot showing the estimated mean change (black dots) and 95% confidence intervals (horizontal bars) for specific time point comparisons (D30 vs. D0, D120 vs. D30, and D120 vs. D0). The vertical dashed red line marks the line of no change (0). For each comparison, the absolute effect size (|eff|) and the respective *p*-value (*p*) are provided.

FACE-Q Neck scores progressively increased over the study period, indicating sustained improvement in patient-reported neck appearance following treatment (Figure 6). The mean scores rose from 48.5 ± 11.0 at baseline to 54.8 ± 14.7 at D30 and 62.5 ± 13.8 at D120, corresponding to mean changes of +6.3 points (D30–D0), +8.3 points (D120–D30), and +14.4 points (D120–D0; Table 6).

The D30–baseline comparison did not reach statistical significance (mean difference = 6.3; 95% CI, −3.2 to 15.9; *p*-value = 0.172), although a small effect size was observed (Cohen’s d = 0.42; Table A4). The D120–D30 contrast similarly showed a numerical increase with a moderate effect size (mean difference = 8.3; 95% CI, −0.5 to 17.1; *p*-value = 0.063; d = 0.63). In contrast, the D120–baseline comparison demonstrated a statistically significant improvement prior to multiplicity correction (mean difference = 14.4; 95% CI, 0.8 to 27.9; *p*-value = 0.039; d = 0.71). However, after Holm adjustment for the three pairwise comparisons, none of the contrasts remained statistically significant (adjusted *p*-value = 0.119 for D120–baseline). Despite the absence of multiplicity-corrected significance, the consistent upward trajectory of scores and the moderate effect sizes observed at later time points support a clinically meaningful trend toward progressive improvement.

### 3.5. STIIM UP Protocol Maintains the Treatment Effect Without Further Improvement

**Figure 7:**
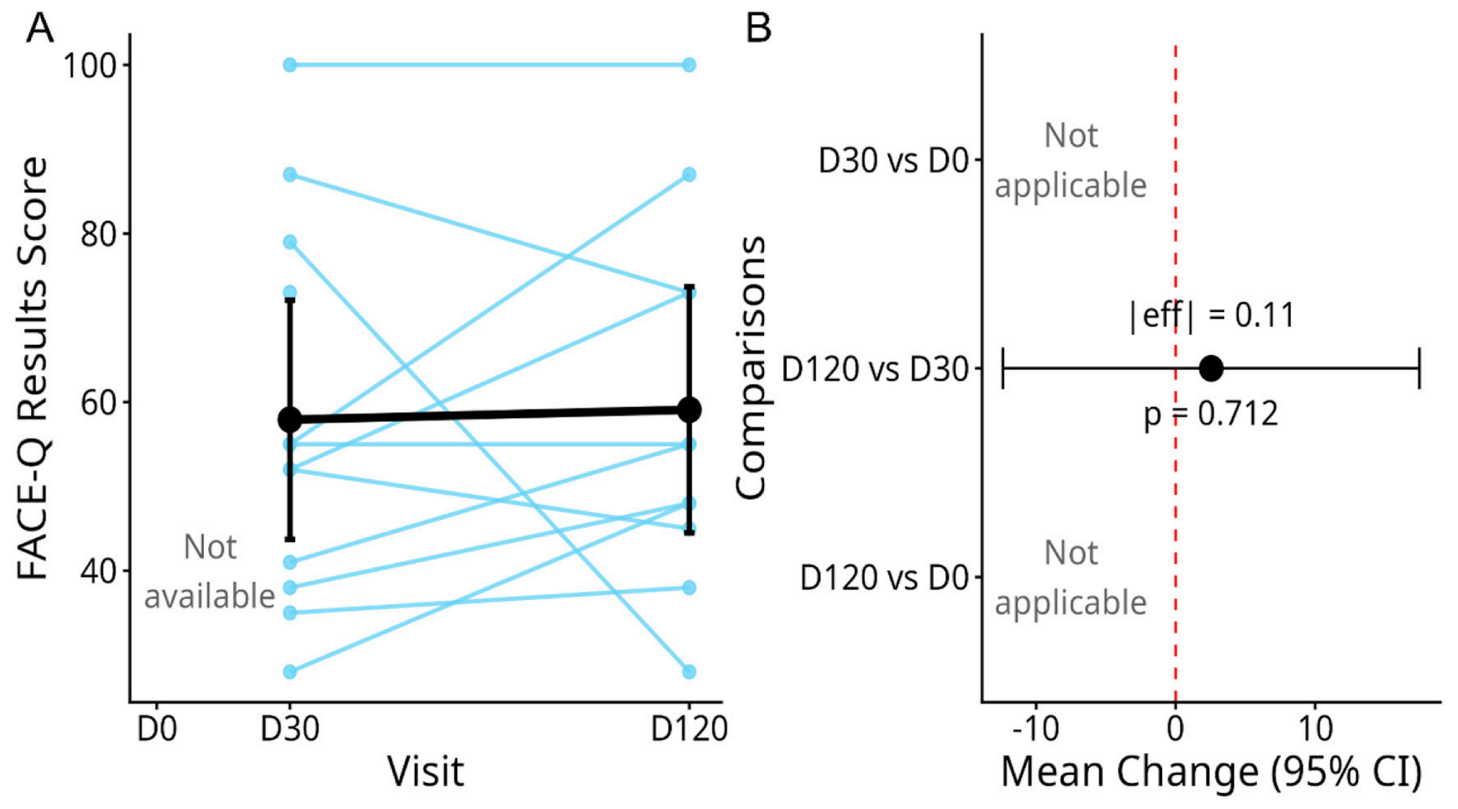
Longitudinal evolution of FACE-Q Satisfaction with Outcome scores between Day 30 and Day 120. (**A**) Individual and mean changes over time. Light blue dots and lines represent individual patient trajectories. The thick black line and error bars represent the mean scores and standard deviations at Day 30 (57.9 ± 22.3) and Day 120 (59.1 ± 21.7). (**B**) Paired comparison and effect size-estimation plot showing the mean difference (black circle) and 95% confidence interval (error bars) for the change between Day 30 and Day 120 (mean difference = 2.5; 95% CI, −12.4 to 17.5). Comparisons involving Day 0 are not applicable.

The FACE-Q Satisfaction with Outcome scores remained largely stable between D30 and D120, with mean values of 57.9 ± 22.3 at D30 and 59.1 ± 21.7 at D120, indicating only a minimal numerical increase over time (Table 7, Figure 7). The paired change between visits was modest (+2.5 points) and characterized by substantial interindividual variability (SD = 22.2), with observed changes ranging from a 51-point decrease to a 32-point increase (Table 7, Figure 7). Consistent with these results, the paired *t*-test revealed no statistically significant difference between D30 and D120 (mean difference = 2.5; 95% CI, −12.4 to 17.5; *p* = 0.712), and the corresponding effect size was negligible (Cohen’s d = 0.11; Table A5, Figure 7). Overall, these findings indicate maintenance of patient-reported outcomes during this interval, without evidence of meaningful improvement or deterioration.

### 3.6. STIIM UP Protocol Increases Epidermal, Dermal, and Subcutaneous Thickness

**Figure 8:**
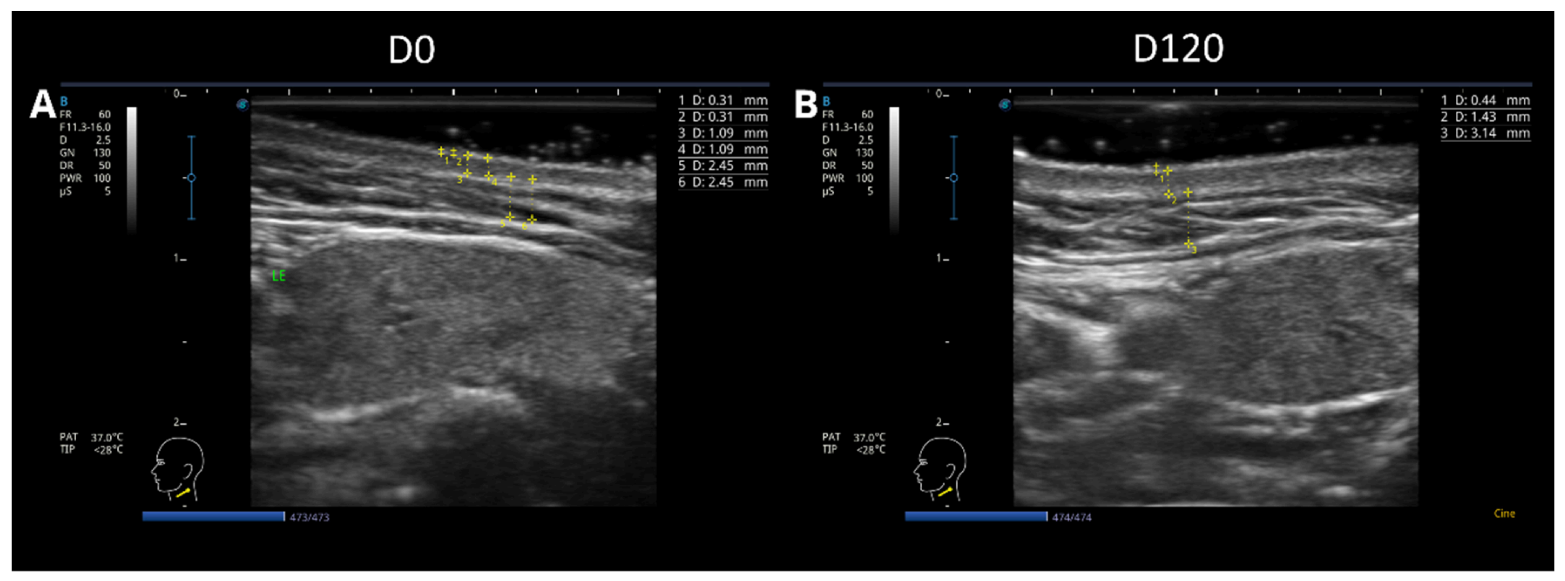
US evaluation of cutaneous and subcutaneous layers before and after treatment with the STIIM UP Protocol. High-resolution ultrasonographic images of the treated region acquired at baseline (D0; (**A**)) and 120 days post-intervention (D120; (**B**)). Linear calipers mark the distinct boundaries of the tissue layers, demonstrating a comprehensive structural reorganization. The yellow “+” symbols indicate the endpoints of the ultrasound calipers used to measure tissue thickness, and the numerical values are reported in millimeters (mm). In panel A, measurements 1–2 correspond to the epidermis, 3–4 to the dermis, and 5–6 to the subcutaneous tissue; in panel B, measurements 1, 2, and 3 correspond to the epidermis, dermis, and subcutaneous tissue, respectively. The alphanumeric information displayed on the left side of each image represents the ultrasound system acquisition and display settings and does not correspond to study measurements or outcomes. Quantitative analysis reveals a substantial increase across all structural layers, with measurements recorded in millimeters (mm): epidermal thickness increased from 0.31 mm at D0 to 0.44 mm at D120; dermal thickness increased from 1.09 mm at D0 to 1.43 mm at D120, reflecting neocollagenesis and extracellular matrix remodeling; and subcutaneous tissue thickness increased from 2.45 mm at D0 to 3.14 mm at D120, demonstrating an overall optimization of the skin bed architecture.

USG analysis at D120 revealed a statistically significant, bilateral increase in tissue thickness across all anatomical strata compared to baseline (D0) (Figure 8).

**Figure 9:**
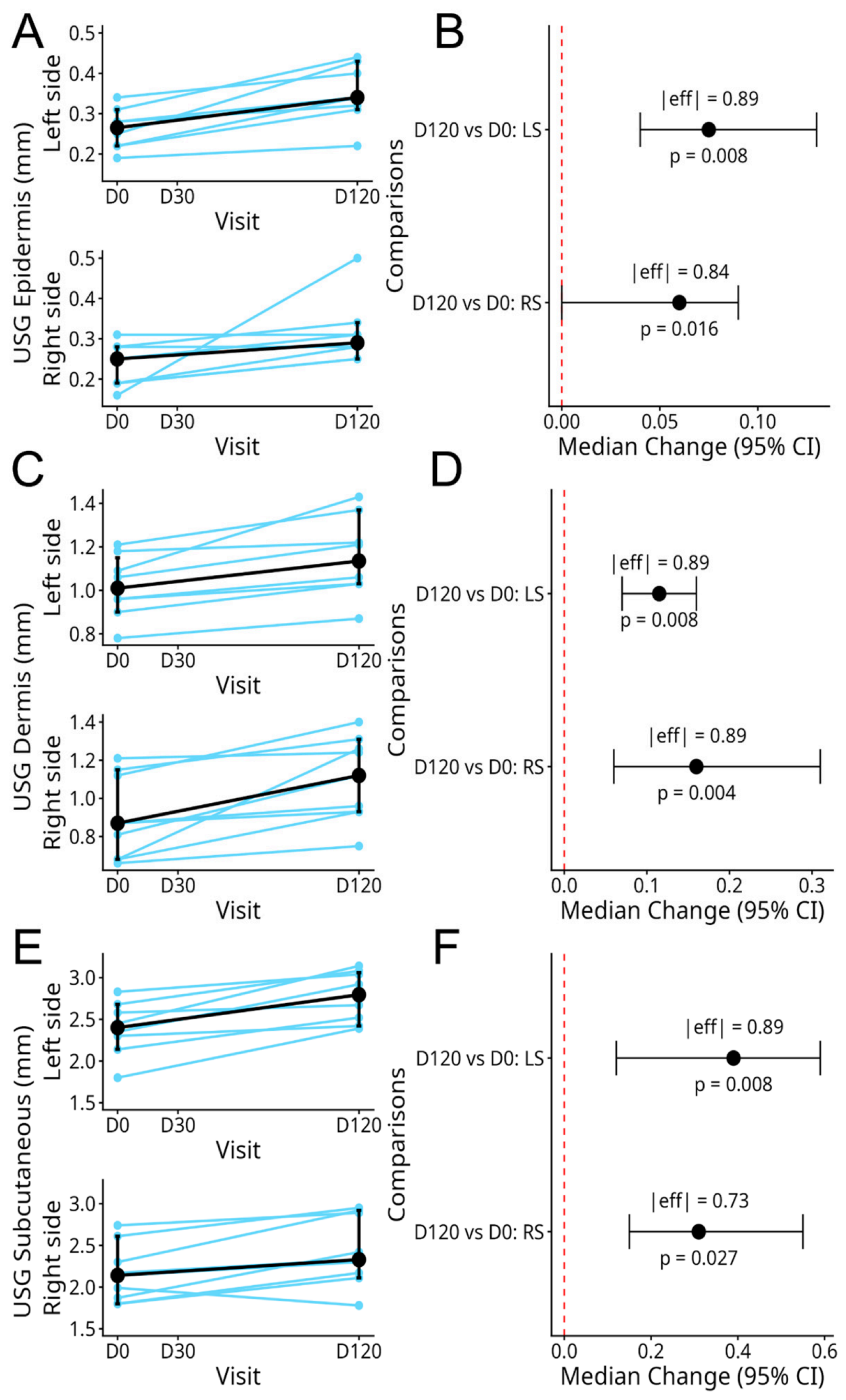
USG longitudinal evaluation of tissue layer thickness from baseline to Day 120. Individual patient trajectories (light blue lines) and overall cohort median shifts with error bars (black lines) illustrate changes in tissue thickness (mm) on both the left and right facial sides of the neck for the (**A**) epidermis, (**C**) dermis, and (**E**) subcutaneous layers. The subcutaneous layer—also known as the hypodermis—is the deepest tissue layer beneath the skin, composed mainly of fat and connective tissue that provides cushioning and structural support. Forest plots show the estimated median differences (delta changes) with bootstrap-derived 95% confidence intervals (CIs) for the (**B**) epidermis, (**D**) dermis, and (**F**) subcutaneous layers. Effect sizes (|eff|, Wilcoxon r) and *p*-values from two-tailed paired Wilcoxon signed-rank tests are provided for each hemispheric comparison (LS: left side; RS: right side). The dashed red vertical line marks the line of no change (zero shift).

Specifically, median epidermal thickness significantly increased on both the left side (from 0.265 mm to 0.340 mm; *p*-value = 0.0078, r = 0.892) and the right side (from 0.250 mm to 0.290 mm; *p*-value = 0.0156, r = 0.844; Figure 9A,B, Table 8). Parallel robust expansions were documented within the dermal compartment, where median thickness increased from 1.01 mm to 1.14 mm on the left side (*p*-value = 0.0078, r = 0.892) and from 0.87 mm to 1.12 mm on the right side (*p*-value = 0.0039, r = 0.894; Figure 9C,D, Table 8). Furthermore, the subcutaneous layer exhibited significant thickening, with median values increasing from 2.40 mm to 2.80 mm on the left (*p*-value = 0.0078, r = 0.892) and from 2.14 mm to 2.33 mm on the right (*p*-value = 0.0273, r = 0.704; Figure 9E,F, Table 8). Delta distribution analyses corroborated these findings, demonstrating consistently positive median shifts across all evaluated compartments, with the most pronounced absolute variations observed in the subcutaneous tissue, thereby confirming a uniform structural remodeling of the cutaneous and soft tissue architecture over the 120-day follow-up period (Table 8).

## 4. Discussion

The clinical management of cervical (neck) aging represents a distinct challenge in esthetic dermatology due to the thinness of the regional dermis and its continuous exposure to dynamic muscular stress. The findings of this prospective clinical trial demonstrate that the STIIM UP Protocol, an innovative, single-session combination of CaHA (STIIM), HA (UP Fine), and BoNT-A, effectively promotes structural remodeling and clinical rejuvenation of the neck. By integrating the long-term neocollagenic properties of CaHA with the immediate skin hydration of HA and the neuromuscular modulating capacity of BoNT-A, this protocol addresses the multifactorial nature of cervical integument degradation in a single, potentially synergistic treatment.

The primary clinical evidence of the protocol’s efficacy is highlighted by the significant reduction in the IBSA Neck Grading Scale scores. The median consensus IBSA scores decreased sharply from 4.0 at baseline to 2.0 at D30, a clinical response that remained stable through to D120. This rapid onset of esthetic improvement can be attributed to the viscoelastic, hygroscopic support provided by HA and the relaxation of the platysma muscle by BoNT-A, which collectively minimize horizontal rhytids and vertical bands. The long-term maintenance of this effect up to D120 is highly indicative of the delayed, progressive biostimulatory activity of CaHA microspheres, which traditionally induce endogenous type I collagen synthesis as the initial carboxymethylcellulose carrier degrades.

Crucially, these clinical observations are supported by the quantitative high-frequency USG findings at the study endpoint. The non-invasive imaging revealed a statistically significant, bilateral increase in thickness across all anatomical strata, including the epidermis, dermis, and subcutaneous layers. The marked expansion of the dermal compartment (with a median increase of 0.12 mm on the left and 0.16 mm on the right) suggests that neocollagenesis and extracellular matrix remodeling were stimulated by the CaHA component. Furthermore, the concurrent thickening of the epidermis and subcutaneous layers points to a tissue-regenerative response, which likely enhances the mechanical properties and barrier function of the thin cervical integument.

From a patient-centered perspective, the protocol demonstrated clinical efficacy, as evidenced by the high satisfaction rates and longitudinal gains recorded in the FACE-Q modules. Although interrater agreement among clinician evaluations and patient self-assessments using the GAIS was low, reflecting the inherent subjectivity of esthetic perceptions, the overall trend was generally favorable, with 100% of evaluators classifying patients as responders and over 90% of patients reporting perceived improvement by D120. The progressive rise in absolute FACE-Q Neck scores underscores a cumulative improvement in appearance-related quality of life. In summary, the STIIM UP Protocol presents a structurally sound, multi-layered approach that achieves comprehensive neck rejuvenation by successfully bridging clinical enhancement associated with improvements in neck appearance measures.

A critical consideration in the chemical management of hyperfunctional platysmal bands is the potential for local diffusion into deeper anatomical structures of the neck. Standalone, deep intramuscular injections of high-dose botulinum toxin type A (BoNT-A) into the platysma muscle bellies carry an inherent risk of vertical tracking through the deep cervical fascia. This diffusion can inadvertently affect the laryngeal musculature or the recurrent laryngeal nerve, clinically manifesting as transient dysphonia (hoarseness) or dysphagia. Crucially, no cases of hoarseness, voice alterations, or swallowing difficulties were observed in our cohort throughout the 120-day follow-up period. This favorable safety profile is directly attributable to the deliberate delivery mechanics of the STIIM UP Protocol. Rather than targeting deep muscular tissues, the hyper-diluted suspension is distributed exclusively within the superficial subdermal plane using a blunt 22G cannula via a retro-injection fanning technique. This superficial placement acts as an anatomical safeguard, minimizing localized deep infiltration and providing a highly secure therapeutic index for comprehensive cervical rejuvenation.

Despite the promising clinical and quantitative outcomes demonstrated by the STIIM UP Protocol, several limitations inherent to this exploratory study must be acknowledged. First, the investigation was conducted as an open-label, single-arm trial with a small sample size (*n* = 13 at baseline), which limits the generalizability of the findings to a broader demographic and precludes direct comparison with a control or monotherapy group. Additionally, participant attrition over the 120-day timeline left only 11 evaluable subjects at the final endpoint. While this sample size is methodologically justified for preliminary safety and efficacy signaling, it lowers the statistical power, as evidenced by the lack of statistical significance in the pairwise longitudinal FACE-Q Neck score contrasts after multiplicity correction. Second, the 120-day (4-month) follow-up period, while sufficient to capture early neocollagenesis and muscle relaxation, is too short to fully determine the long-term durability and structural degradation rate of the combined suspension. Lastly, the subjective efficacy scales (GAIS and IBSA) revealed notable interrater variability, particularly the GAIS, which exhibited negligible to weak concordance among independent evaluator evaluations and patient self-ratings. This underscores the challenges of relying on subjective esthetic appraisals and highlights the necessity for larger, randomized, controlled clinical trials with extended follow-up periods to definitively establish protocol longevity and optimize candidate selection.

## 5. Conclusions

In conclusion, the exploratory clinical trial evaluating the STIIM UP Protocol demonstrates that the single-session, synergistic combination of CaHA, HA, and BoNT-A provides an effective and safe approach for comprehensive neck rejuvenation. The protocol achieved a rapid, clinically meaningful reduction in cervical skin laxity by D30 that was successfully maintained through to the 120-day endpoint. These macroscopic, scale-based improvements were objectively validated by high-frequency ultrasound analysis, which documented significant bilateral tissue thickening across the epidermis, dermis, and subcutaneous strata, confirming robust multi-layered structural remodeling. Additionally, the intervention yielded high rates of patient-reported satisfaction and a favorable safety profile with no observed adverse effects. Taken together, these preliminary findings suggest that the STIIM UP Protocol represents a promising therapeutic strategy to address the multifactorial nature of cervical aging, though larger randomized controlled trials are warranted to confirm its long-term durability and optimize patient selection.

## Figures and Tables

**Table 1:** Baseline demographic and clinical characteristics of study population.

Attribute		Statistics	Cohort Analysis (*n* = 13)	
**Age (years)**		mean, sd	48.45	7.45
**Gender**				
	Female	*n*, %	13	100%
**Ethnicity**				
	Brown	*n*, %	1	8%
	Not Reported		1	8%
	White		11	85%
**Weight (kg)**		mean, sd	56.58	7.89
**Height (cm)**		mean, sd	159.38	6.56
**BMI (kg/m^2^)**		mean, sd	22.22	2.04
**Medical history**				
	None	*n*, %	9	69%
	Other		1	8%
	Smoking		2	15%
	Smoking/Hypertension		1	8%
**Previous procedures**				
	None	*n*, %	9	69%
	Yes		4	31%

**Table 2:** Descriptive analysis of IBSA scores.

Attribute	Visit	Statistics	Values		95% CI
IBSA score	D0	Median, IQR	4	2.0	[3; 5]
	D30		2	1.0	[2; 3]
	D120		2	0.5	[2; 3]

**Table 3:** Statistical inference of time variation in IBSA score.

Attribute	Comparison	Statistics	Δ		95% CI	*p*-Value	*p*-Adj	r
IBSA	D30 vs. D0	Median, IQR	−2	1	[−2; −1]	0.0009	0.0029	0.89
	D120 vs. D30		0	1	[−1; 0]	0.0625	0.0625	0.67
	D120 vs. D0		−2	1	[−3; −1]	0.0009	0.0029	0.90

**Table 4:** IBSA responder analysis.

Comparison	Responders	Proportion	95% Exact CI
D30 vs. D0	11/12	91.7%	[61.5%; 99.8%]
D120 vs. D30	5/11	45.5%	[16.7%; 76.6%]
D120 vs. D0	11/11	100%	[71.5%; 100%]

Responders: ≥1-point IBSA improvement.

**Table 5:** GAIS responder analysis.

Rater	Comparison	Responders	Proportion	Exact 95% CI
Evaluator 1	D30	12/12	100%	[73.5%; 100%]
	D120	11/11	100%	[71.5%; 100%]
Evaluator 2	D30	12/12	100%	[73.5%; 100%]
	D120	11/11	100%	[71.5%; 100%]
Evaluator 3	D30	12/12	100%	[73.5%; 100%]
	D120	11/11	100%	[71.5%; 100%]
Patient	D30	10/12	83.3%	[51.5%; 97.9%]
	D120	10/11	90.9%	[58.7%; 99.8%]

**Table 6:** Descriptive analysis of FACE-Q-Neck scores.

Visit	Statistics	Values		95% CI
D0	Mean, sd	48.5	11.0	[41.5; 55.5]
D30		54.8	14.7	[45.5; 64.2]
D120		62.5	13.8	[53.3; 71.8]

**Table 7:** Descriptive analysis of FACE-Q Satisfaction with Outcome scores.

Visit	Statistics	Values		95% CI
D0	Mean, sd	(−)	(−)	(−)
D30		57.9	22.3	[43.7; 72.1]
D120		59.1	21.7	[44.5; 73.7]

**Table 8:** Quantitative USG tissue thickness measurements and comparative intra-subject delta (Δ) distribution analysis between baseline (D0) and Day 120 (D120).

Attribute	Comparison	Statistics	Δ (mm)		95% CI	*p*-Value	Eff. Size
**Epidermis**		Median, IQR					
*LS*	D120 vs. D0		0.08	0.07	[0.04; 0.13]	0.008	0.89
*RS*			0.06	0.02	[0; 0.09]	0.016	0.84
**Dermis**		Median, IQR					
*LS*	D120 vs. D0		0.12	0.07	[0.07; 0.16]	0.008	0.89
*RS*			0.16	0.19	[0.06; 0.31]	0.004	0.89
**Subcutaneous**		Median, IQR					
*LS*	D120 vs. D0		0.39	0.39	[0.12; 0.59]	0.008	0.89
*RS*			0.31	0.21	[0.15; 0.55]	0.027	0.73

LS—left side; RS—right side.

## Data Availability

All data supporting the findings of this study are available within the article. No additional datasets are available.

## Appendix A

**Table A1:** Agreement among evaluators on the ordinal IBSA scale.

IBSA Concordance Cohen’s Kappa				
Visit	Raters	Kappa	*p*-Value	*n*
D0	Evaluator 1 vs. 2	0.295	0.037	12
	Evaluator 1 vs. 3	0.360	0.028	12
	Evaluator 2 vs. 3	0.952	0.001	12
D30	Evaluator 1 vs. 2	0.333	0.182	12
	Evaluator 1 vs. 3	0.556	0.026	12
	Evaluator 2 vs. 3	0.063	0.805	12
D120	Evaluator 1 vs. 2	0.522	0.022	11
	Evaluator 1 vs. 3	0.790	0.002	11
	Evaluator 2 vs. 3	0.607	0.037	11

**Table A2:** Agreement among evaluators on the GAIS.

Visit	Raters	Kappa	*p*-Value	*n*
D0	Evaluator 1 vs. 2			
	Evaluator 1 vs. 3			
	Evaluator 2 vs. 3			
D30	Evaluator 1 vs. 2	0.108	0.687	12
	Evaluator 1 vs. 3	−0.014	0.961	12
	Evaluator 2 vs. 3	0.093	0.734	12
D120	Evaluator 1 vs. 2	−0.303	0.248	11
	Evaluator 1 vs. 3	0.175	0.511	11
	Evaluator 2 vs. 3	−0.069	0.712	11

**Table A3:** Agreement among patients and evaluators on the GAIS.

Visit	Patient vs.	Kappa	*p*-Value	*n*
D0	Evaluator 1			
	Evaluator 2			
	Evaluator 3			
D30	Evaluator 1	0.148	0.595	12
	Evaluator 2	−0.141	0.622	12
	Evaluator 3	−0.530	0.060	12
D120	Evaluator 1	0.245	0.121	11
	Evaluator 2	0.015	0.946	11
	Evaluator 3	0.038	0.742	11

**Table A4:** Statistical inference of time variation in FACE-Q Neck score.

Comparison	Statistics	Δ		95% CI	*p*-Value	*p*-Adj	Eff. Size
D30 vs. D0	Mean, sd	6.33	15.0	[−3.22; 15.9]	0.1723	0.1720	0.42
D120 vs. D30		8.27	13.1	[−0.55; 17.1]	0.0632	0.1260	0.63
D120 vs. D0		14.4	20.1	[0.84; 27.9]	0.0395	0.1185	0.71

**Table A5:** Statistical inference of time variation in FACE-Q Satisfaction with Outcome score.

Comparison	Statistics	Δ		95% CI	*p*-Value	*p*-Adj	Eff. Size
D30 vs. D0	Mean, sd	-	-	-	-	-	-
D120 vs. D30		2.55	22.2	[−12.4; 17.5]	0.7121	0.7121	0.11
D120 vs. D0		-	-	-	-	-	-
